# Nuclear and mitochondrial genomes of the plum fruit moth *Grapholita funebrana*

**DOI:** 10.1038/s41597-024-03522-7

**Published:** 2024-06-26

**Authors:** Li-Jun Cao, Fangyuan Yang, Jin-Cui Chen, Shu-Jun Wei

**Affiliations:** Institute of Plant Protection, Beijing Academy of Agriculture and Forestry Sciences, Beijing, China

**Keywords:** Genomics, Agricultural genetics

## Abstract

The plum fruit moth *Grapholita funebrana* (Tortricidae, Lepidoptera) is an important pest of many wild and cultivated stone fruits and other plants in the family Rosaceae. Here, we assembled its nuclear and mitochondrial genomes using Illumina, Nanopore, and Hi-C sequencing technologies. The nuclear genome size is 570.9 Mb, with a repeat rate of 51.28%, and a BUCSO completeness of 97.7%. The karyotype for males is 2n = 56. We identified 17,979 protein-coding genes, 5,643 tRNAs, and 94 rRNAs. We also determined the mitochondrial genome of this species and annotated 13 protein-coding genes, 22 tRNAs, and 2 rRNA. These genomes provide resources to understand the genetics, ecology, and genome evolution of the tortricid moths.

## Background & Summary

The plum fruit moth *Grapholita funebrana* is an important fruit borer from the family Tortricidae of Lepidoptera^1,2^. Larvae of *G. funebrana* cause damage by boring the fruits of many wild and cultivated stone fruits and other plants in the family Rosaceae, such as apricot, cherry, peach, and plum^3^. This species is native to Europe and currently found in fruit-growing regions of Europe, northern Africa, and Asia^4^. In the orchards, *G. funebrana* often co-occur with other fruit borers, such as the oriental fruit moth *Grapholita* *molesta* (Busck), the codling moth *Cydia pomonella*, and peach fruit moth *Carposina sasakii* Matsumura^5^. While many studies have focused on the biology and management of fruit borers, research on *G. funebrana* is lagging behind^6–10^. In addition, moths from the family Tortricidae are ideal for unveiling the evolution of chromosome fusion^11,12^. While species from the order Lepidoptera often have a conserved chromosome number of n = 31, in the Tortricidae family, many species have a reduced number of chromosomes due to the fusion of chromosome pairs^13,14^. Recent research has found that a common ancestor of the suborders Tortricinae and Olethreutinae diverged from the ancestral lepidopteran chromosome pattern due to a fusion of sex chromosomes with autosomes^15^. The karyotype of tortricid moths was traditionally studied by cytogenetic methods and fluorescence *in situ* hybridization^15^. Determining the genome sequences will improve understanding of the molecular evolution of chromosomes of tortricid moths^16^. Currently, chromosome-level genomes have been published for the *C. pomonella*^16^, and *G. molesta*^17^, as well as many publicly available assemblies for Tortricidae in the GenBank (https://www.ncbi.nlm.nih.gov/datasets/genome/?taxon=7139).

In this study, we assembled a chromosome-level genome for the *G. funebrana* as well its mitochondrial genome using Oxford Nanopore Technologies (ONT) long-read sequencing, Illumina short-read sequencing, high-throughput chromatin conformation capture (Hi-C) sequencing, and RNA-sequencing (RNA-seq). We yielded a nuclear genome assembly of 570.9 Mb, with an N50 of 21 Mb. These high-quality genomes will provide invaluable resources for the study of *G. funebrana* and in-depth investigation of chromosome evolution on macroevolutionary and microevolutionary levels.

## Methods

### Material and sequencing

Apricot (*Prunus armeniaca*) fruits with *G. funebrana* larvae were collected from Yanqing, Beijing, China, and reared in the laboratory for about 30 days to obtain specimens of different developmental stages. To decrease the effect of heterozygosity, a single larva was used for long-read, short-read, and Hi-C library construction. Single larva, pupa, and adult (unknown sex) were collected for the construction of RNA-seq libraries, respectively. All samples were immediately flash-frozen in liquid nitrogen and stored at −80 °C for subsequent experiments.

Genomic DNA was extracted using the Magnetic bead method (Invitrogen, Thermo Fisher Scientific, USA), while RNA was extracted using RNAprep Pure Plus Kit (Tiangen, China), respectively. The quantity of DNA was measured using Qubit 3.0. To generate short-read data for the genome survey, an Illumina library with an insert size of 350 bp was constructed and sequenced on the Illumina NovaSeq 6000 platform. To perform *de novo* genome assembly, a 15~20 kb ONT library was prepared and sequenced on the ONT platform to generate long-read data. To generate the Hi-C data, tissue from a larva was fixed with paraformaldehyde and digested with restriction enzymes *DnpII*, generating fragments with sticky ends. These sticky ends were repaired using DNA polymerase and ligated together to form chimeric circles using DNA ligase. The ligated DNAs were then decrosslinked, purified, and sheared into 350 bp insertion size. The Hi-C sequencing library was sequenced on the Illumina NovaSeq 6000 platform to generate 150-bp paired-end reads. Paired-end libraries were constructed using the VAHTSTM mRNA-seq V2 Library Prep Kit (Vazyme, Nanjing, China) and then sequenced on the Illumina NovaSeq 6000 platform with PE reads of 150 bp for genome annotation. A total of 33.7 Gb Illumina short read, 69.7 Gb ONT long-read, 58.3 Gb Hi-C reads, and 21.9 Gb RNA-seq reads data were generated. The raw data of Illumina reads were filtered by Fastp v0.21.0^18^ with default parameters.

### Genome survey

Genome survey was performed using a k-mer based method. The k-mer coverage was counted from Illumina short reads using Jellyfish version 2.2.10^19^ with parameters: ‘count -m 21 -C -s 5 G’. Genome size, heterozygosity, and duplication rate were estimated using GenomeScope version 2.0^20^. The results showed a genome size about 515 Mb, a heterozygosity rate of 1.91%, and a duplication rate of 1.21%.

### Genome assembly

**Fig. 1:**
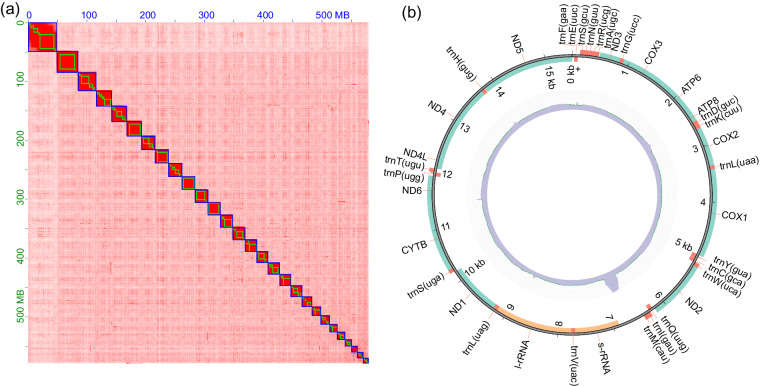
The interaction heat map of nuclear genome (**a**), and distribution of genes and read coverage on mitochondrial genome (**b**).

The Nanopore long reads were assembled to the primary set of nuclear genome contigs using NextDenovo v2.5.1^21^ with parameters: ‘read_cutoff = 1k, genome_size = 400 m, pa_correction = 20, nextgraph_options = -a 1’. The contigs contain 215 sequences, with a size of 594 Mb, and N50 of 6.6 Mb. Due to the high error rate of assembly based on ONT reads, the primary contigs were polished using NextPolish 1.4.1^22^ with one round based on long reads and one round based on short reads. To achieve chromosome-level assembly, the polished contigs were anchored into pseudomolecules based on Hi-C reads information. Specifically, the Hi-C reads were mapped to contigs using Chromap 0.2.4^23^ with options: “–preset hic–remove-pcr-duplicates–trim-adapters–SAM”. The SAM output was sorted by read name and output to BAM format using Samtools v1.17^24^ with options: “sort -n -O BAM”. Yahs v1.2a.1^25^ and Juicerbox 1.22.01^26^ were then used for unsupervised and supervised scaffolding, respectively. After scaffolding, most contigs (95.3% contigs and 99.86% base-pairs) were anchored into 28 pseudo-chromosomes (Fig. 1a), consistent with the karyotype of most species in the subfamily Olethreutinae. To fill the gaps between contigs, we performed two rounds of polishing based on long- and short-reads using Nextpolish. The final assembly has a genome size of 570.9 Mb, with a N50 of 21 Mb. The assembled genome is 56.9 Mb larger than the estimated genome size. MitoZ v3.6 pipeline^27^ was performed to assembly using Megahit v1.29^28^ (“–kmers_megahit 39 59 79 99 119 141–requiring_taxa Lepidoptera”) and annotate mitochondrial genome. The mitochondrial genome of *G. funebrana* was 15,488 bp in length and contain 13 protein coding genes, 22 tRNA genes and 2 rRNA genes (Fig. 1b).

### Genome annotations

For repeat sequence annotation, a species-specific repeat library was generated using RepeatModeler v2.0.4^29^ with options: “-LTRStruct”. The species-specific repeat library, a RepBase database, and a repeat element library for Arthropoda from the Dfam database were then combined and passed to RepeatMasker v4.1.4^30^ for repeat annotation. RepeatMasker was performed with options:” -no_is -norna -xsmall -q”.

For gene structure annotation, we performed a pipeline integrating RNA-seq-based, *ab initio*, and homolog-based methods. The RNA reads of single larva, pupa and adult libraries were mapped to our final assembly with Hisat v2.2.0^27^ and assembled to transcripts with Stringtie v2.1.2^31^. The transcriptome assemblies and protein sequences of *Plutella xylostella* (Accession: GCA_932276165.1^32^) were provided as evidence to MAKER v3.01.04 pipeline^26^ to integrate. SNAP v2013-02-16^28^ and Augustus v3.2.3^29^ were used to conduct *ab initio* annotation. Transfer RNA (tRNA) was predicted using tRNAscanSE 2.0.12^33^ with default parameters, and ribosome RNA (rRNA) was predicted using Barrnap 0.9 (https://github.com/tseemann/barrnap). The above gene models were merged to produce consensus models by EvidenceModeler v2.1.0^33^. Functional annotation of protein-coding genes was evaluated using EggNOG-mapper v2^34^.

### Chromosome feature

**Fig. 2:**
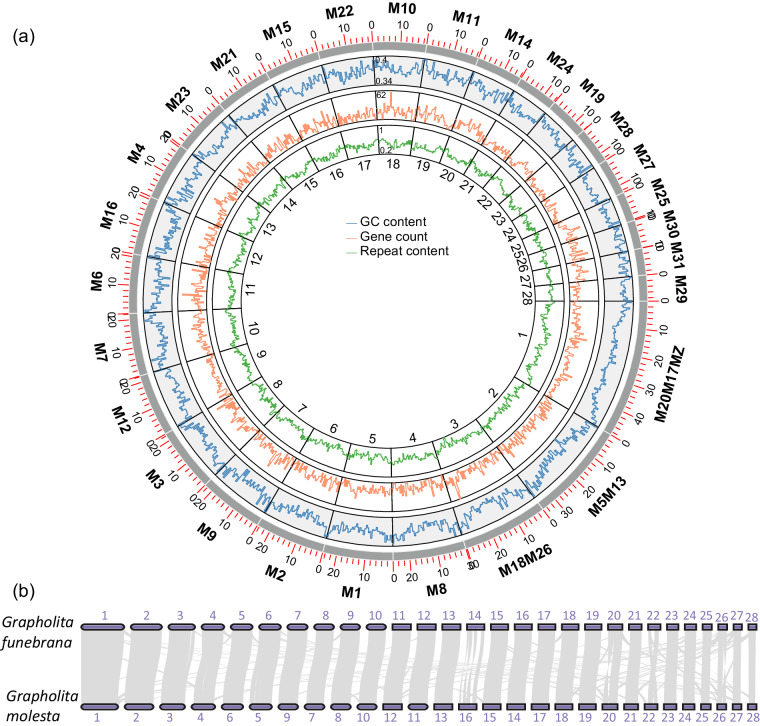
Chromosome features of *Grapholita funebrana* genome. (**a**) Circos plot of GC content, gene count, and repeat content. Chromosomes were labeled using Merian elements according to the homology with the Lepidopteran ancestral linkage groups^14^. (**b**) Synteny blocks between the *G. funebrana* and *G. molesta* reveal the same number of chromosomes and highly conserved gene order in the two moths. The chromosomes of two genomes were numbered according to their length. The grey lines show the synteny blocks between two genomes.

The gene number, repeat sequence density, and Guanine-Cytosine(GC) content were calculated in 500 Kb non-overlapping sliding windows using Bedtools v2.30.0^35^. The name of the chromosomes was assigned as lepidopteran ancestral linkage groups^14^, based on homology to *Sesia bembeciformis*^36^. The homology was detected using LAST^37^ alignment. A Circos plot of chromosome feature was generated by TBtools v2.021^38^ (Fig. 2a).

## Data Records

Illumina, Nanopore, Hi-C, and transcriptome data for *G. funebrana* genome sequencing have been deposited in the NCBI Sequence Read Archive with accession number SRP482231^39^. The final assembled nuclear genome of *G. funebrana* has been deposited in the NCBI Genbank with accession number GCA_038095595.1^40^. The mitochondrial genome has been deposited in the NCBI Genbank with accession number PP776023^41^. The genome assembly and annotation files are available in Figshare^42^.

## Technical Validation

The Hi-C heatmap revealed a well-structured interaction pattern. Short-read sequencing data were mapped to the final assembly with BWA v0.7.17^43^, revealing a mapping rate of 97.7%. The completeness of *G. funebrana* genome assembly was evaluated using the BUSCO^44^ base on the lepidoptera_odb10 database (n = 5286). The completeness of the initial assembly (contig level) was 90.9%, while it increased to 97.7% (97.2% single-copied genes, 0.5% duplicated genes, 0.6% fragmented, and 1.7% missing genes) after polishing with NextPolish^22^ (Table 1). We identified 14,547 protein-coding genes, 11,673 of which were functionally annotated. The completeness of the annotated gene set was 95.8% (94.8% single-copied genes and 1.0% duplicated genes, 1.1% fragmented, and 3.1% missing genes). A synteny analysis between *G. funebrana* and *G. molesta*^17^ was performed using MCSCAN in JCVI package^45^. Strong syntenic blocks were found between the two closely related species (Fig. 2b). All evidence strongly supported the completeness and accuracy of *G. funebrana* genome assembly.

**Table 1:** Statics of *G. funebrana* genome assembly.

Item	Contig	Purged contig	Hi-C raised scaffold	Polished scaffold
No. of contigs	215	175	28	28
Size (Mb)	593.9	580.3	579.6	570.9
N50 (Mb)	6.6	7.2	21.4	21.0
GC content	37.8%	37.6%	37.6%	37.5%
Single-copy BUSCOs	90.2%	90.9%	90.5%	97.2%
Duplicated BUSCOs	0.7%	0.4%	0.3%	0.5%
Fragmented BUSCOs	4.4%	4.4%	4.4%	0.6%
Missing BUSCOs	4.7%	4.7%	4.8%	1.7%

## Data Availability

No custom scripts or code were used in this study.

## References

[1] Li L-L (2023). Functional disparity of four pheromone-binding proteins from the plum fruit moth *Grapholita funebrana* Treitscheke in detection of sex pheromone components. Int. J. Biol. Macromol..

[2] Lo Verde G, Guarino S, Barone S, Rizzo R (2020). Can mating disruption be a possible route to control plum fruit moth in mediterranean environments?. Insects.

[3] Dickler, E. Tortricid pests of pome and stone fruits, eurasian species. in *Tortricids Pests, Their Biology, Natural Enemies and Control* (eds. van der Geest, L. P. S. & Evenhuis, H. H.) 435–452 (Elsevier, Amsterdam, Netherlands, 1991).

[4] F, K. A taxonomic review of the genus *Grapholita* and allied genera (Lepidoptera: Tortricidae) in the Palaearctic region. *Ent. Scand. Suppl*. **55**, 110 (1999).

[5] Chen MH, Dorn S (2009). Reliable and efficient discrimination of four internal fruit-feeding *Cydia* and *Grapholita* species (Lepidoptera: Tortricidae) by polymerase chain reaction-restriction fragment length polymorphism. J. Econ. Entomol..

[6] Ioriatti C (2009). Toxicity of emamectin benzoate to *Cydia pomonella* (L.) and *Cydia molesta* (Busck) (Lepidoptera: Tortricidae): laboratory and field tests. Pest Manag. Sci..

[7] Liu J (2022). Reverse chemical ecology guides the screening for *Grapholita molesta* pheromone synergists. Pest Manag. Sci..

[8] Stelinski LL, Il’ichev AL, Gut LJ (2009). Efficacy and release rate of reservoir pheromone dispensers for simultaneous mating disruption of codling moth and oriental fruit moth (Lepidoptera: Tortricidae). J. Econ. Entomol..

[9] Witzgall P, Stelinski L, Gut L, Thomson D (2008). Codling moth management and chemical ecology. Annu. Rev. Entomol..

[10] Wu Y (2022). Laboratory evaluation of the compatibility of *Beauveria bassiana* with the egg parasitoid *Trichogramma dendrolimi* (Hymenoptera: Trichogrammatidae) for joint application against the oriental fruit moth *Grapholita molesta* (Lepidoptera: Tortricidae). Pest Manag. Sci..

[11] Nguyen P (2013). Neo-sex chromosomes and adaptive potential in tortricid pests. Proc. Natl. Acad. Sci..

[12] Sahara K, Yoshido A, Traut W (2012). Sex chromosome evolution in moths and butterflies. Chromosome Res..

[13] Nguyen, P. & Carabajal Paladino, L. On the neo-sex chromosomes of Lepidoptera. in *Evolutionary Biology: Convergent Evolution, Evolution of Complex Traits, Concepts and Methods* (ed. Pontarotti, P.) 171–185. 10.1007/978-3-319-41324-2_11 (Springer International Publishing, Cham, 2016).

[14] Wright, C. J., Stevens, L., Mackintosh, A., Lawniczak, M. & Blaxter, M. Comparative genomics reveals the dynamics of chromosome evolution in Lepidoptera. *Nat. Ecol. Evol*. 1–14, 10.1038/s41559-024-02329-4 (2024). 10.1038/s41559-024-02329-4 PMC11009112 38383850

[15] Šíchová J, Nguyen P, Dalíková M, Marec F (2013). Chromosomal evolution in tortricid moths: conserved karyotypes with diverged features. PLoS ONE.

[16] Wan F (2019). A chromosome-level genome assembly of *Cydia pomonella* provides insights into chemical ecology and insecticide resistance. Nat. Commun..

[17] Cao L-J (2022). Population genomic signatures of the oriental fruit moth related to the Pleistocene climates. Commun. Biol..

[18] Chen S, Zhou Y, Chen Y, Gu J (2018). fastp: an ultra-fast all-in-one FASTQ preprocessor. Bioinformatics.

[19] Marçais G, Kingsford C (2011). A fast, lock-free approach for efficient parallel counting of occurrences of k-mers. Bioinformatics.

[20] Vurture GW (2017). GenomeScope: fast reference-free genome profiling from short reads. Bioinformatics.

[21] Hu J (2024). NextDenovo: an efficient error correction and accurate assembly tool for noisy long reads. Genome Biol..

[22] Hu J, Fan J, Sun Z, Liu S (2020). NextPolish: a fast and efficient genome polishing tool for long-read assembly. Bioinformatics.

[23] Zhang H (2021). Fast alignment and preprocessing of chromatin profiles with Chromap. Nat. Commun..

[24] Danecek P (2021). Twelve years of SAMtools and BCFtools. GigaScience.

[25] Zhou C, McCarthy SA, Durbin R (2023). YaHS: yet another Hi-C scaffolding tool. Bioinformatics.

[26] Durand NC (2016). Juicer provides a one-click system for analyzing loop-resolution Hi-C experiments. Cell Syst..

[27] Meng G, Li Y, Yang C, Liu S (2019). MitoZ: a toolkit for animal mitochondrial genome assembly, annotation and visualization. Nucleic Acids Res..

[28] Li D, Liu C-M, Luo R, Sadakane K, Lam T-W (2015). MEGAHIT: an ultra-fast single-node solution for large and complex metagenomics assembly via succinct de Bruijn graph. Bioinformatics.

[29] Flynn JM (2020). RepeatModeler2 for automated genomic discovery of transposable element families. Proc. Natl. Acad. Sci..

[30] Tarailo-Graovac M, Chen N (2009). Using RepeatMasker to identify repetitive elements in genomic sequences. Curr. Protoc. Bioinforma..

[31] Pertea M, Kim D, Pertea GM, Leek JT, Salzberg SL (2016). Transcript-level expression analysis of RNA-seq experiments with HISAT, StringTie and Ballgown. Nat. Protoc..

[32] (2022). Genbank.

[33] Chan PP, Lin BY, Mak AJ, Lowe TM (2021). tRNAscan-SE 2.0: improved detection and functional classification of transfer RNA genes. Nucleic Acids Res..

[34] Cantalapiedra CP, Hernández-Plaza A, Letunic I, Bork P, Huerta-Cepas J (2021). eggNOG-mapper v2: Functional annotation, orthology assignments, and domain prediction at the metagenomic scale. Mol. Biol. Evol..

[35] Quinlan AR (2014). BEDTools: The swiss-army tool for genome feature analysis. Curr. Protoc. Bioinforma..

[36] (2022). Genbank.

[37] Katoh K, Frith MC (2012). Adding unaligned sequences into an existing alignment using MAFFT and LAST. Bioinformatics.

[38] Chen C (2020). TBtools: An Integrative Toolkit Developed for Interactive Analyses of Big Biological Data. Mol. Plant.

[39] (2024). NCBI Sequence Read Archive.

[40] (2024). Genbank.

[41] (2024). Genbank.

[42] Wei S-J, Yang F (2024). Figshare.

[43] Li H, Durbin R (2009). Fast and accurate short read alignment with Burrows–Wheeler transform. Bioinformatics.

[44] Manni M, Berkeley MR, Seppey M, Simão FA, Zdobnov EM (2021). BUSCO update: Novel and streamlined workflows along with broader and deeper phylogenetic coverage for scoring of eukaryotic, prokaryotic, and viral genomes. Mol. Biol. Evol..

[45] Tang H (2008). Synteny and collinearity in plant genomes. Science.

